# Modern family planning use among people living with HIV/AIDS: a facility based study in Ethiopia

**DOI:** 10.11604/pamj.2019.33.224.19025

**Published:** 2019-07-18

**Authors:** Akateh Derek, Assefa Seme, Cho Sabastine Anye, Claude Ngwayu Nkfusai, Samuel Nambile Cumber

**Affiliations:** 1 Department of Public Medicine and Surgery, Faculty of Health Sciences, University of Buea, Buea, Cameroon; 2 Department of Reproductive Health and Health System Management, School of Public Health, Addis Ababa University, Addis Ababa, Ethiopia; 3 Cameroon Baptist Convention Health Services (CBCHS), Yaoundé, Cameroon; 4 Department of Microbiology and Parasitology, Faculty of Science, University of Buea, Buea, Cameroon; 5 Faculty of Health Sciences, University of the Free State, Bloemfontein, South Africa; 6 Institute of Medicine, Department of Public Health and Community Medicine (EPSO), University of Gothenburg, Box 414, SE-405 30 Gothenburg, Sweden; 7 School of Health Systems and Public Health, Faculty of Health Sciences, University of Pretoria Private Bag X323, Gezina, Pretoria, 0001, Pretoria, South Africa

**Keywords:** Family planning, PLWHA, ART-clinics, socio-cultural factors, Addis Ababa, Ethiopia

## Abstract

**Introduction:**

Despite increasing efforts to address the reproductive health needs of persons living with Human Immuno-Deficiency Virus (HIV), a high unmet need for contraception exists among HIV+ women in sub-Saharan Africa. Currently, Ethiopia promotes integration of family planning (FP) services in to HIV chronic care. Yet the contraceptive prevalence rate among clients remains low. The objective of the study was to assess the role of socio-cultural factors on modern family planning use among HIV+ clients attending Anti-Retroviral Therapy clinics in Addis Ababa sub-cities.

**Methods:**

The study involved a facility based cross sectional survey. The ten sub cities were initially categorized/stratified into 5 based on direction (East, West, South, North and Central) and from each category one sub city was randomly selected. The total sample size was proportionally allocated to the selected health facilities according to previous monthly average client load per health center. Participants were selected using simple random sampling technique during their routine visit at the health centers. Data were collected through a semi-structured interviewer administered questionnaire. Both descriptive and inferential statistics were generated and results considered significant at 95% confidence level using STATA version 14.0.

**Results:**

Six hundred and thirty-six clients participated in the study. Majority of them were age between 30-39 years. Though majority, 607 (95.4%) participants approved the use of modern FP method, current use rate stood at 39%. Condom was the most (14.5%) commonly used single method. The odds of FP use by participants who disclosed their HIV status were almost twice that of their counterparts (AOR= 1.84; 95% CI: 1.14, 2.95). Participants who held discussion with their spouse/partners concerning FP, irrespective of the frequency had an odd of more than four when using FP than their counterparts (AO= 4.35; 95% CI: 2.69, 7.04).

**Conclusion:**

This study revealed that 6 out of every 10 HIV+ clients are not currently using FP methods. Disclosure of HIV status as well as open discussion with spouse/partner were positively associated with family planning use. These study findings call for comprehensive and client focus FP education and counseling in line with disclosure of HIV status and dialogue with spouse/partner in order to increase uptake and utilization of FP among clients. Partners have a great influence on the use and choice of FP methods, so their views are paramount.

## Introduction

Globally, there are an estimated 36.7 million people living with HIV/AIDS. The pandemic burden lies in Africa where it disproportionally affects Sub-Sahara Africa (SSA) inhabitants. Greater than half (58%) of persons living with HIV are of reproductive age and slightly more than half (53%) of all adult deaths is related to HIV in this region [1]. Despite increasing efforts to address the reproductive health needs of people living with HIV, 80% unmet need for family planning (FP) exists among HIV+ clients in sub-Saharan Africa [2]. HIV positive clients who do not use modern FP have a higher risk of unwanted pregnancies and hence increase chance of MTCT [3] and unsafe abortion. Ethiopia, annually expects 3 million pregnancies of which 602 births per 100,000 pregnancies are prone to MTCT of HIV with a large majority of this pregnancies been unwanted [4]. A cross-sectional study conducted in Ethiopia including both HIV-positive and HIV-negative women reported that 69.2% of their most recent pregnancy was unwanted [5]. Besides the benefits in averting HIV positive births and preventing unwanted pregnancies, FP is also a cost effective strategy. For instance, providing family planning to HIV positive women in Ethiopia is expected to save annually US $360,000 than providing ARV prophylaxis [6]. Undesired pregnancies have also both maternal and child consequences including unsafe abortion, maternal and infant morbidity and mortality.

The Ethiopian national plan for the elimination of MTCT of HIV is in line with the global comprehensive PMTCT strategies. It advocates the four pronged strategies to keep the mother alive and prevent new pediatric HIV infections [7]. The four pillars of the strategy are primary prevention of HIV infection, preventing unintended pregnancy among HIV-infected women, preventing HIV transmission from HIV-infected women to their infants, and care of HIV-infected women and their children [8]. In Ethiopia currently, the Ministry of Health (MOH) policies promote integration of FP services in HIV/AIDS prevention, care and treatment services [8]. Yet a large number (65-72%) of HIV positive women still have unintended (unwanted or mistimed) pregnancies [9]. Several studies conducted in many African countries have indicated that individual, reproductive, societal expectation, medical intervention (Highly Active Anti-Retroviral Therapy [HAART] and Prevention of Mother to Child Transmission [PMTCT]), health services, and cultural and belief factors influence family planning use among HIV positive clients [10-16]. Socio-cultural factors like disclosure of HIV status to spouse [17], open discussion about family planning method and use [12], decision making about family planning use [13, 18], as well as partner support and approval of FP method use were found to be positively associated with current family planning use. However, there are limited studies conducted in Ethiopia following literature reviews that have looked into socio-cultural factors influencing Modern family planning utilization among People Living with HIV/AIDS (PLWHA); an area advocated by many researchers to explore. Hence, the aim of this study was to address the knowledge gap with regards to factors influencing family planning use among PLWHA, in a bid to help program managers and stakeholders better plan for this target population in terms of decision making and policy development. This study, therefore, sought to assess socio-cultural factors that influence modern family planning utilization in the context of ART service provision among sexually active PLWHA enrolled at governmental health centers of Addis Ababa.

## Methods

**Study area and** period: the study was conducted in Addis Ababa, the capital of Ethiopia. Its population is totally urban with population projection of 3,048,632 million; 1,595,968 females in 2017 [6]. Addis Ababa City Administration is made up of 10 sub cities and 116 woredas harboring 52 Hospitals (public and private owned), 84 Health Centers (80 government owned and 4 by NGOs) and more than 760 health clinics from low to higher (all Private) [19]. There are 112 ART sites in the city; 75 health centers, 8 NGOs facilities, 11 government hospitals and 18 private hospitals [19]. Public and private health facilities are offering ART services in the city since the free ART lunched in 2005 in Ethiopia [20]. In 2015, in Addis Ababa, there were 129,143 people who have ever started ART and 82,498 currently on treatment [3]. In 2014, 42% of married women of reproductive age (15-49) were using FP methods [7]. Amharic is a commonly spoken language in the study area. This study was conducted at health centers having an ART clinic providing FP services in five randomly selected sub-cities in Addis Ababa from August 2016 to June 2017.

**Study design:** this is a facility based cross-sectional study design aimed to gather data of PLWHA who were seeking services from Addis Ababa City Administration health centers. Medical chart/file was reviewed to confirm the HIV status of each participant before inviting to participate in the study.

**Eligibility criteria:** to be eligible to participate in the study, participants were sexually active PLWHA; female and male aged 18-49 years who accepted to participate in the study and were able to give informed consent; attending the randomly selected health centers ART clinics during the study period. We considered a participant to be ART user if he/she was taking ART at the time of interview.

**Sample size:** the sample size was calculated using single population proportional formula. We assume proportion of modern FP use among PLWHA (both males and females)= 50%= 0.5 (No study to the knowledge of the authors report proportion of FP use among both male and female HIV sero-positive clients and also needed the largest possible sample size for a cross sectional study); Absolute precision of 5% and 95% level of confidence yielding 384 participants. Due to the multi-stage random sampling nature of the study design, a design effect of 1.5 was used giving a minimum sample of 578 participants. Assuming a 10% non-response rate, the final sample size for the study was 636 participants.

**Figure 1:**
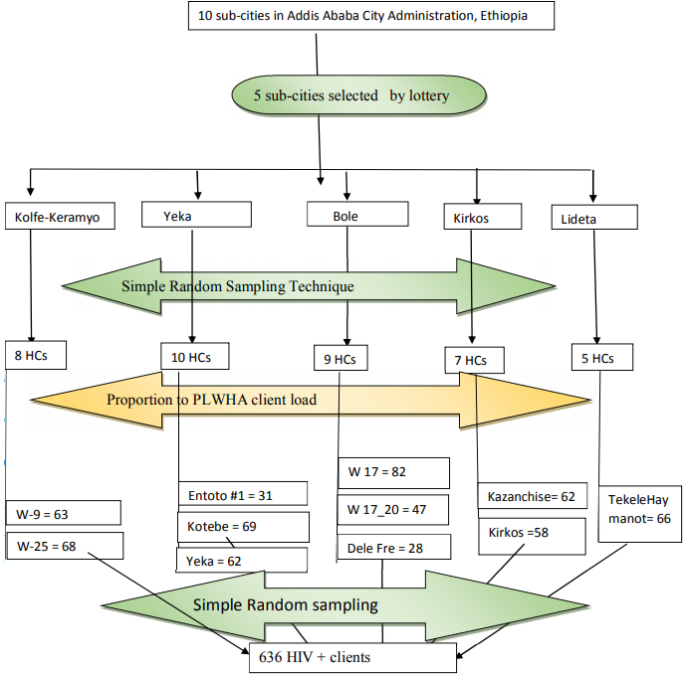
schematic presentation of sampling procedure

**Sample selection:** study participants were selected using multi-stage random sampling technique. Addis Ababa has 10 sub-cities hosting 75 health centers (HCs). Fifty percent of these sub-cities were selected with simple random sampling technique using lottery method after been stratified into north, west, east, south and central. All HCs in the selected sub-cities providing ART services were listed. The numbers of HCs in each selected sub-city were determined proportionally considering the total number of HCs in each sub-city. Eleven HCs were selected. The numbers of study participants in each HC was determined using proportion to population size where the total sample size was proportionally allocated to the selected HCs according to client load (the number of PLWHA receiving ART services) in each facilities to meet up the study sample size (Figure 1). At the level of the HCs, participants were selected using simple random sampling; knowing the HIV codes of all the patients, a random selection was done and the selected clients were interview on a daily basis as they came to pick their medications after ensuring they meet the inclusion criteria stated above. It was repeated until the sample size of each facility was reached. If the client does not meet or is not willing to participate or a sample client does not show up to pick up his/her ARV, the client next to him/her was sampled and enrolled for the study.

**Data collection procedures and enumerators:** the interview was conducted during the routine health facility visits. Invitation to participate in the study was done during the short brief health talks organized in each health facilities prior to commencement of routine health facilities activities. This was done in collaboration with respective health facilities providers. After confirming eligibility and seeking written informed consent, participants were asked to complete a 25-30 minutes structured interviewer-administered questionnaire in Amharic (a local language). Approximately 4-6 clients were interviewed daily. Data collectors were trained staff from each health facilities ART clinic who had previous research experience to maintain confidentiality and obtain genuine responses. Interviews were conducted from April to May 2017. Questionnaires were filled under strict confidentiality.

**Data collection instruments:** the survey instrument was adapted from ones used by other authors and modified following literature review of articles [9, 12, 13, 18, 21]. The questionnaire was used to collect information on socio-demographic characteristic, knowledge, source of information on FP methods, attitude and use of modern FP, fertility history, their HIV-status and that of their spouse/partner, socio-cultural and beliefs factors influencing the utilization of modern FP methods. The questionnaire was developed in English and later translated into Amharic. Consistency and accuracy check was done to ensure proper and correct translation of the questions through back translation so that the meaning of the question is not lost. The questionnaire was pre-tested before it rollout for the study (Figure 1).

**Measures and operational definitions:** the primary outcome was self-reported contraceptive use at the time of the survey. *Current use of FP method* refers to respondents who responded positively after being asked whether they were currently doing anything to delay or avoid pregnancy. The time period for current use of FP varies; For surgical methods such as female sterilization (tubal ligation) and male sterilization (vasectomy) - ever use will assess as these are permanent FP methods; For methods such as oral contraceptives, injectable, intrauterine contraceptive device (IUD), implants- their current contraceptive effect at the time of the interview will assess as this effect is temporary; For barrier methods such as condoms-current use was a reported use by sexually active PLWHA for FP purposes or for any other reason at the time of the interview irrespective of the consistency. In assessing the contraceptive method profile, dual protection was defined as use of both a barrier contraceptive method (male condom) and use of a hormonal or permanent contraceptive method [22]. *Ever used FP method-* any participant who said has used a modern FP method before and who is either using or not using at the time of the interview. *FP services-* a health services that help individuals and couples decide whether to have children and if so, when and how many, and to achieve the desired spacing and timing of their births. *Contraceptive Prevalence rate-* Is the proportion of women of reproductive age who are using (or whose partner is using) a modern FP method at a given point in time. *Sexual intercourse-* was defined to refer specifically to vaginal-penile penetrative sex between a man and a woman. *Sexually Active-* A client who has had sexual intercourse at least once in the last 3 months. We define ART user as any PLWHA who is receiving ART in the study facilities at the time of interview.

**Data quality control, management and analysis:** first, codes were given to the completed questionnaires and then data were entered into Epi-data Version 3.5.1, 2008 (CDC, Atlanta, Georgia) database and transferred to STATA version 14 (Stata Corporation, College Station, TX, USA) statistical packages for analysis. Data collectors were fluent in the local language with a degree in Nursing and training was given to them on the importance of research and techniques/skills on interview, sampling and ethical issues, emphasizing the importance of safety of participants and interviewers, minimization of under reporting and maintaining confidentiality. Pretest of questionnaire was conducted out of study health centers on 5% of the sample size. Supervisors had checked filled questionnaires and checklists for consistency, completeness and accuracy on daily base for all clients. Supportive supervision by the principal investigator was also rendered for each data collector and supervisor in process. Cleaning of data was done to check the consistency and completeness of the data set.

Frequencies were calculated for categorical variables and summary measures for continuous variables to describe the study population in relation to relevant variables. Tables and graphs were used to present the data. Descriptive statistics was used to show the frequency and percentage of the characteristics. Both bivariable and multivariable logistic regression analyses were used to identify significant predictors. The degree of association between independent and dependent variables was assessed using odds ratio with 95% confidence interval and p-values were obtained. The finding at this stage was to help identify important associations. After testing for co-linearity and interaction, all factors with statistically significant associations with FP use in the bivariable analysis (p<0.05); and those not significant but with previous evidence from literature review indicating possible association with FP use were considered in the multivariate logistic regression model (Forward stepwise model). The adjusted estimates of the association between covariates and family planning use was obtained. Their respective adjusted odds ratios (AOR) associated with these potential factors were reported as a measure of strength, together with the respective 95% confidence intervals.

**Ethical consideration:** ethical approval and ethical clearance was obtained from Research and Ethics Committee of the School of Public Health, Addis Ababa University and permission letter was obtained from Addis Ababa City Administration Health Bureau. Support letters were sent to various health facilities managers for permission to carry out the research. Furthermore, during the data collection, written informed consent was obtained from each respondent by first explaining the objectives and content of the study. Some of the study participants were unable to read and write. In this case, trained interviewers fully explained the purpose, process, benefits, and risks to all study participants before consent was obtained and inked index finger print was used as a signature. The information collected was purely used for research works and name of respondents remained anonymous. All interviews were conducted in a private room. To maintain anonymity identifiers like names were not captured in the questionnaire. All measures to maintain human subjects including informed consent; the right to participate in the study, right to privacy and confidentiality and right to prevention from any type of harm was taken into consideration. All participants were informed that their participation is on voluntarism bases and they have the right to stop or withdraw should they feel discomfort during the interview. The interview lasted no more than 30 minutes per participants. Data were kept confidential by locking in a cupboard with key and by password in the computer to avoid access of the data by a third party.

## Results

**Socio-demographic characteristics of the study participants:** a total of 636 sexually active respondents aged between 20 to 49 years participated in the study. Females made up 86.2% of the study participants. The mean age (±SD) of respondents was 33.9 (±6.2) years. Majority 386 (60.1%) of the respondents were found equally in the age ranges 30-34 and 35-39 years (Table 1). Of the 636 respondent, 362 (57.2%) were either married or cohabiting. Most 264 (41.5%) of respondents were orthodox. Concerning spouse/partner educational level, most 597 (93.8%) had attended at least a formal education of which secondary educational level accounted for 42.45%. Respondent educational level showed similar profile to that of their spouse/partner. Occupationally, most 300 (47.2%) of the spouse/partner were daily laborers while majority 194 (30.5%) of the respondents were private business owners. About two-thirds 439 (69.1%) of the respondents had at least a biological child with the median number of living children being 1 (IQR= 0,6, Min 0, Max 8) (Table 1).

**Table 1:** socio-demographic characteristics of PLWHA of reproductive age group in Addis Ababa city region, Ethiopia, 2017

Characteristic	Categories	Number (n)	Percentage (%)
**Sex**	Male	88	13.8
	Female	548	86.2
**Age at interview in years**	20-24	36	5.7
	25-29	94	14.8
	30-34	193	30.3
	35-49	193	30.3
	40-44	83	13.1
	45-49	37	5.8
**Marital status**	Single	59	9.3
	Married/Cohabited partner	362	56.9
	Separated/Divorced	9	1.4
	Widowed/ widower	206	32.4
**Spouse/partner Educational level**	Illiterate	20	3.1
	Primary	105	15.5
	Secondary	270	42.5
	Technical/vocational	133	21.9
	Tertiary and above	89	14.9
	Didn’t know spouse/partners educational level	19	2.1
**Educational level**	Illiterate	6	1.0
	Primary	161	25.3
	Secondary	287	45.1
	Technical/vocational	108	17.0
	Tertiary and above	74	11.6
**Religion**	Orthodox	264	41.5
	Muslim	73	11.5
	Catholic	185	29.1
	Protestant	114	17.9
**Occupation of spouse/partner**	Unemployed	24	3.8
	Private business/merchant	113	17.8
	Farmers	67	10.5
	Government employee	81	12.7
	Daily laborer	300	47.2
	Housewife	15	2.4
	Others	36	5.6
**Occupation**	Unemployed	119	18.7
	Private business/merchant	194	30.5
	Farmers	65	10.2
	Government employee	57	9.0
	Daily laborer	27	4.3
	Housewife	149	23.4
	Others	25	3.9
**Biological children**	Yes	439	69.0
	No	197	31.0
**Total # of living children respondents have**	None	197	30.9
	1	170	26.8
	2	171	26.9
	3+	98	15.4

**Figure 2:**
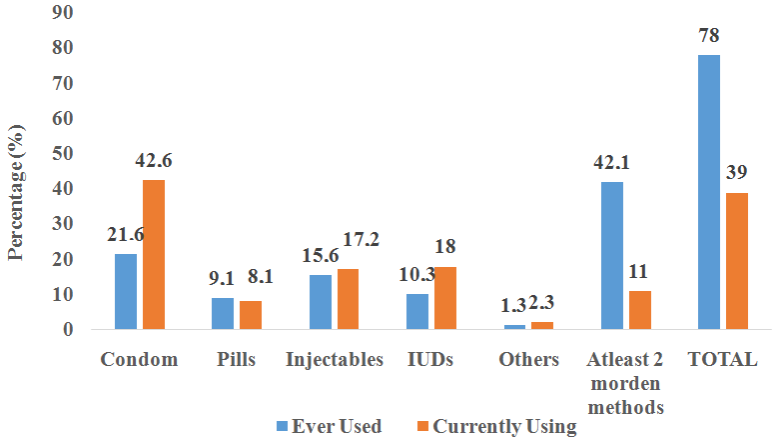
FP methods used at the time of survey and ever used among PLWHA of reproductive age group in Addis Ababa city, Ethiopia, 2017

**Family planning use and methods preference:** of the 636 respondents, 607 (95.4%) participants approved the use of modern FP methods. Five hundred and four (79.2%) respondents or their partners had ever used modern FP methods. Among all the respondents, 248 (39%) were currently using modern FP. Family planning methods preferences are shown in Figure 2. As shown, the most common type of single birth control method ever used were condoms 109 (21.6%), injections 79 (15.6%), IUD 52 (10.3%) and oral pills 46 (9.1%). Less than half 213 (42.1%) of this cohort had ever used at least 2 modern FP methods. Among current contraceptive users, the number of condoms users was high (42.6%), followed by IUD (18%). Twenty-eight (11%) participants used at least two modern FP methods during the survey (dual method) (Figure 2). Half of the participants 123 (49.7%) using modern FP during the survey had a history of FP usage. In general, long acting (implants) and permanent methods were least known and had the same frequency of usage in the ‘ever’ and ‘current’ use status (figure not shown).

**Association of socio-cultural variables with utilization of modern family planning methods:** in bivariate analysis, disclosure of HIV status to spouse/partner, having an open discussion about FP methods and their usage, as well as decision making about FP usage had a crude significant association with use of modern FP methods(p<0.05). Also, partner/spouse approval on FP methods was significantly associated with FP usage. Close relations and peers did not influence FP usage. In contrast, not having a male child significantly influenced FP usage, with many respondents not using any method due to their search for a male child. Traditional and religious beliefs as well as cultural norms/values were not significantly associated with FP usage (Table 2). Using a forward step wise multivariable logistic regression model and controlling for all other socio demographic variables (age, sex, education, residence, income), disclosure of HIV status to spouse/partner and having an open discussion about FP with spouse/partner showed a significant positive association with modern FP use among PLWHA (p<0.05) (Table 2). Participants who disclosed their HIV status to their spouse/partner and jointly made decision on FP use had almost two times the odds (AOR= 1.84; 95% CI: 1.14, 2.95) and four times the odds (AOR= 4.35; 95% CI: 2.69, 7.04) respectively of using a modern FP method. The other factors which were significantly associated with modern FP usage in the bivariate analysis lost their association in the multivariable analysis.

**Table 2:** multivariable forward stepwise logistic regression model predicting current FP use among PLWHA

Characteristic	Current use of modern FP		COR (95%CI)	AOR (95%CI)	p-value
	YES	NO			
**Disclosure of HIV status to Spouse/partner**					
Yes	217(45.2)	263(54.8)	2.78(1.83,4.23)**	1.84(1.14,2.95)	0.012*
No	35(22.9)	118(77.1)	**1(ref)**		
**Discussion with husband/partner about FP methods and use**					
Yes	211(52.1)	194(47.9)	5.01(3.37,7.47)**	4.35(2.69,7.04)	0.000**
No	39(17.8)	180(82.2)	**1 (ref)**		
**Decision making about FP use**					
Jointly	150(47.3)	167(52.7)	1.59(1.12,2.27)*	1.14(0.75,1.72)	0.08
Partner/spouse only	24 (36.4)	66(63.6)	0.64(0.37,1.11)	0.59(0.33,1.08)	0.07
Me only	78(36.1)	138(63.9)	**1 (ref)**		
**Partner/spouse approve use of modern FP**					
Yes	201(48.9)	210(51.1)	3.38(2.21,5.18)*	1.62(0.96,2.73)	0.07
No/ I don’t know	51(47.9)	169(52.1)	**1(ref)**		
**Close relations influence FP use**					
Yes	124(45.8)	147(54.2)	1.51(1.00,2.26)	1.26(0.78,2.03)	0.342
No/ I don’t know	128(35.5)	233(64.5)	**1(ref)**		
**Peer influence on FP use**					
Yes	69(44.5)	86(55.5)	**1(ref)**		
No/ I don’t know	182(38.2)	294(61.8)	1.34(0.88,2.09)	1.16(0.95,3.02)	0.510
**Not having a male child influence on FP use**					
Yes	49(36.3)	86(63.7)	**1 (ref)**		
No	203(40.5)	298(59.5)	1.59(1.00,2.58)*	1.97(0.48,3.47)	0.447
**Traditional beliefs influence FP use**					
Yes	174(39.7)	264(60.3)	1.03(0.72,1.47)	1.05(0.70,1.60)	0.805
No/ I don’t know	81(40.9)	117(59.1)	**1 (ref)**		
**Religious belief favors FP use**					
Yes	118(41.1)	169(58.9)	**1 (ref)**		
No/ no sure	133(38.1)	216(61.9)	0.93(0.66,1.31)	0.91(0.60,1.39)	0.668
**Cultural norms/values supports FP use**					
Yes	118(38.7)	187(61.3)	0.87(0.62,1.23)	1.72(0.98,3.07)	0.115
No/ I don’t know	134(41.9)	197(58.1)	**1 (ref)**		

COR-Crude Odds Ratio, AOR-Adjusted Odds Ratio, 1= reference category.

* significant at P= 0.05

** significant at P<0.001.

## Discussion

The current study was done to assess the role of socio-cultural factors on modern family planning use among HIV+ clients attending Anti-Retroviral Therapy clinics in Addis Ababa sub-cities. In the study, 39% of PLWHA used modern family planning at the time of the survey. This contraceptive prevalence rate (CPR) among this group was low compared to the target set by the Federal HIV/AIDS Prevention and Control Office (FHAPCO) of Ethiopia that target modern FP use among sexually active clients as 50% [5]. The study showed a similarly low FP uptake with a study carried out in Bahir-Dar Town, Northwest Ethiopia where 44.3% of the respondents used FP at the time of the survey [13]. This low FP use can be attributed to inadequate information pertaining to the appropriate choice of a FP method as well as misconceptions about FP. This information inadequacy could also partially explained why fear of side effects (mostly not absolute side effect) of FP methods was the main reason mentioned by those not using any FP methods at the time of the survey. This knowledge gap could also be the reason why respondents were novice to the use of some long acting FP methods like implants and any permanent FP methods. Low levels of FP utilization by PLWHA has been reported to be associated with high rate of unintended pregnancy, vertical and sexual transmission of the virus, leading to an increase in level of abortions, morbidity and mortality [1, 3, 9]. Consequences of low FP uptake was not investigated in our study. This low usage could be improved through an urgent need of making various methods available, as well as the education of PLWHA so that they can use any of the modern FP methods [22] and more specifically counseling on the true side effects of each method. However, some studies done in Uganda and South Africa reported a high proportion of use 78% [23] and 89.8% [24] respectively compared to ours. This high levels of usage could be due to the high level of integration of FP services with HIV/AIDS prevention, care and treatment services in these countries. Suggestively, the high burden of the disease in the aforementioned countries had probably made health education a cornerstone of all disease programs [18].

Findings from a cross-sectional study done in Baringo North District in Kenya was slightly lower than ours (32.3%) [18]. This could have been a result of the difference in the study time, the method acceptance among the two societies, the weakness of the health care services as well as the nature of the study area (subsistence farming rural community). Addis Ababa being an urban setting, participants probably had more exposure to information via TV and radios where knowledge and awareness about FP could be obtained as well as easy access to Health facilities compared to the rural Baringo community. Respondents who disclosed their HIV status to spouse/partner were more likely to use FP than their counterparts. This result was consistent with reports from other studies which stated that ‘respondents who did not disclose their HIV status to their spouse/partners where less likely to use FP methods’ [13, 20, 23]. This is because HIV is nowadays less seen as a taboo in many societies and stigmatization has reduced among most communities [23]. HIV+ clients are freer to disclose their HIV status to spouse/partner leading to communication about health and fertility desires among couples and gaining spousal/partner support. This encourages the use of FP to prevent unwanted pregnancies and the transmission of the virus to offspring. Disclosure of status also brings about partner and family support to the use of FP and a better understanding of some of the myths and misconceptions surrounding the use of FP by PLWHA, thereby increasing their use. Discussions with spouse/partner about FP methods and use was found to be positively associated with current FP use. Studies have reported that discussion with spouse/partner about FP is associated with increased FP use among HIV positive women of reproductive age [20, 24].

Findings from this study revealed that compared to those respondents who did not discuss about FP with their spouse/partners, those who had discussions, irrespective of the frequency had more than four times the odds of using modern FP method. This study indicates that couple discussion about FP method and use positively influence its use and male partner involvement in FP decision remains an important component of FP usage. Interpretation of these results should be within the framework of the following limitations. Because of the cross sectional nature of this study, it's impossible to draw definitive conclusions on causal inference between explanatory factors and FP usage. We can only discuss association in terms of plausibility. Because of fear of stigmatization, participant's responses may have been biased and result may not be generalizable to the entire population of PLWHA on ART care. Another limitation to this study was the lack of qualitative methods which could help explore in-depth views about the role of socio-cultural factors on the use of FP methods. As to the strengths of this study, the respondents have been selected by random sampling technique with relatively large sample size making it representative of the study area. Experienced data collectors who were involved in collection of similar data were selected for this study.

## Conclusion

This study revealed a low used of modern FP among PLWHA most especially the utilization of the permanent methods. From the current study we can conclude that disclosure of one's HIV status to a spouse/partner and having discussion with spouse/partners about family planning, has positively influenced family planning use among the study participants. Health care providers should encourage HIV+ or ART clients to disclose their HIV status to their partner/spouse to gain support including support to use FP. Also, they should encourage HIV+ or ART clients to initiate or strengthen discussion about FP with their spouses/partners. The role of health care providers in providing counselling to materialize the aforementioned is indispensable. Also, government policies and strategies in relation to family planning practices should pay more emphasis on male involvement, availability and information about long acting FP methods in HIV/AIDS clinics. In addition, further research into qualitative assessment of cultural and belief factors on non-use of FP among HIV clients on chronic care and psychosocial determinants of FP use is proposed.

### What is known about this topic

- Barriers to contraceptive use among child bearing women;
- Contraceptive utilization and associated factors among HIV positive women on chronic follow up care;
- Priorities for family planning and HIV/AIDS integration, maximizing access and quality (MAQ) initiative.

### What this study adds

- This study revealed a low used of modern FP among PLWHA most especially the utilization of the permanent methods;
- From the current study we can conclude that disclosure of one's HIV status to a spouse/partner and having discussion with spouse/partners about family planning, has positively influenced family planning use among the study participants

## Competing interests

The authors declare no competing interests.
